# Early‐onset interstitial pneumonitis in a patient with advanced non‐small cell lung cancer treated with crizotinib and osimertinib

**DOI:** 10.1111/1759-7714.13785

**Published:** 2021-01-12

**Authors:** Yuan Cheng, Qing Yu, Yan Xiong, Cuiyan Guo, Ligong Nie

**Affiliations:** ^1^ Department of Respiratory and Critical Care Medicine Peking University First Hospital Beijing China; ^2^ Department of Pathology Peking University First Hospital Beijing China

**Keywords:** Crizotinib, early‐onset interstitial pneumonitis, NSCLC, Osimertinib

## Abstract

Both crizotinib and osimertinib have been reported to have an adverse effect of interstitial pneumonitis in the treatment of non‐small cell lung cancer (NSCLC). Here, we report the case of a 60‐year‐old male patient with advanced NSCLC resistant to osimertinib. Crizotinib was administered in combination with osimertinib due to elevated mesenchymal epithelial transition (MET) copy number amplification. However, early‐onset interstitial pneumonitis occurred within two days.

## Abstract 2

Both crizotinib and osimertinib had an adverse effect of interstitial pneumonitis in non‐small cell lung cancer (NSCLC) treatment. Here, we report a 60‐year‐old male patient with advanced non‐small cell lung cancer resistant to osimertinib. Crizotinib was used in combination with osimertinib due to elevated mesenchymal epithelial transition (MET) copy number amplification, but an early‐onset interstitial pneumonitis occurred within two days.

## Introduction

Mesenchymal epithelial transition (MET) amplification is one of the mechanisms of epidermal growth‐factor receptor tyrosine kinase inhibitor (EGFR‐TKI) acquired resistance. As a MET inhibitor, the efficiency of crizotinib in the treatment of MET amplification has been confirmed. Most patients tolerate crizontinib and osimertinib well. Interstitial pneumonitis is a rare and serious adverse effect with tyrosine kinase inhibitor (TKI) treatment, but the mortality rate is high once it occurs. The currently reported risk factors for EGFR‐TKI‐related interstitial lung disease (ILD) include age, being male, a previous history of interstitial lung disease, and complicated heart disease. A small sample of retrospective research data reported that the combined use of the two drugs did not increase the incidence of side effects.^1^ Here, we report a case of early‐onset interstitial pneumonia following the combined use of crizontinib and osimertinib.

## Case report

In May 2017, a 60‐year‐old male patient, who presented with a cough, was admitted to our hospital. His chest computed tomography (CT) scan in May 2017 had revealed an opacity in the left lung. CT‐guided lung biopsy and PET/CT results confirmed a diagnosis of adenocarcinoma with multiple bone metastases. This patient had no history of smoking or pulmonary disease.

**Figure 1:**
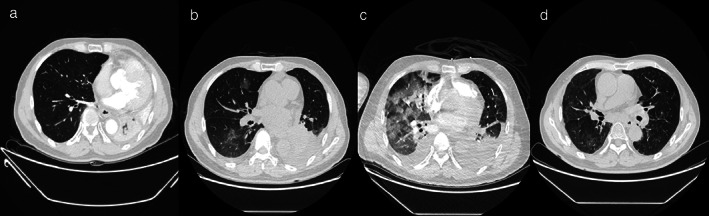
(**a**) CT scan shows left lung atelectasis. (**b**). CT scan shows multiple mild diffuse ground‐glass opacity (GGO) in the right lung after two days of crizotinib and osimertinib administration. (**c**) CT scan shows multiple severe diffuse GGO in both lungs after seven days of crizotinib and osimertinib treatment. (**d**) CT scan taken six weeks later shows diffuse GGO absorbed after glucocorticoid therapy.

**Figure 2:**
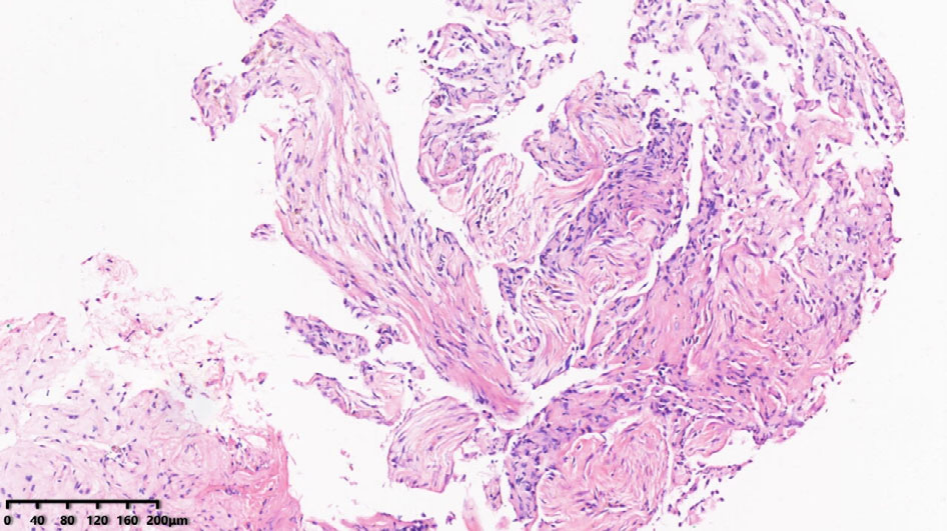
Organizing pneumonia in transbronchial lung biopsy of the right lung (HE, HPF).

**Figure 3:**
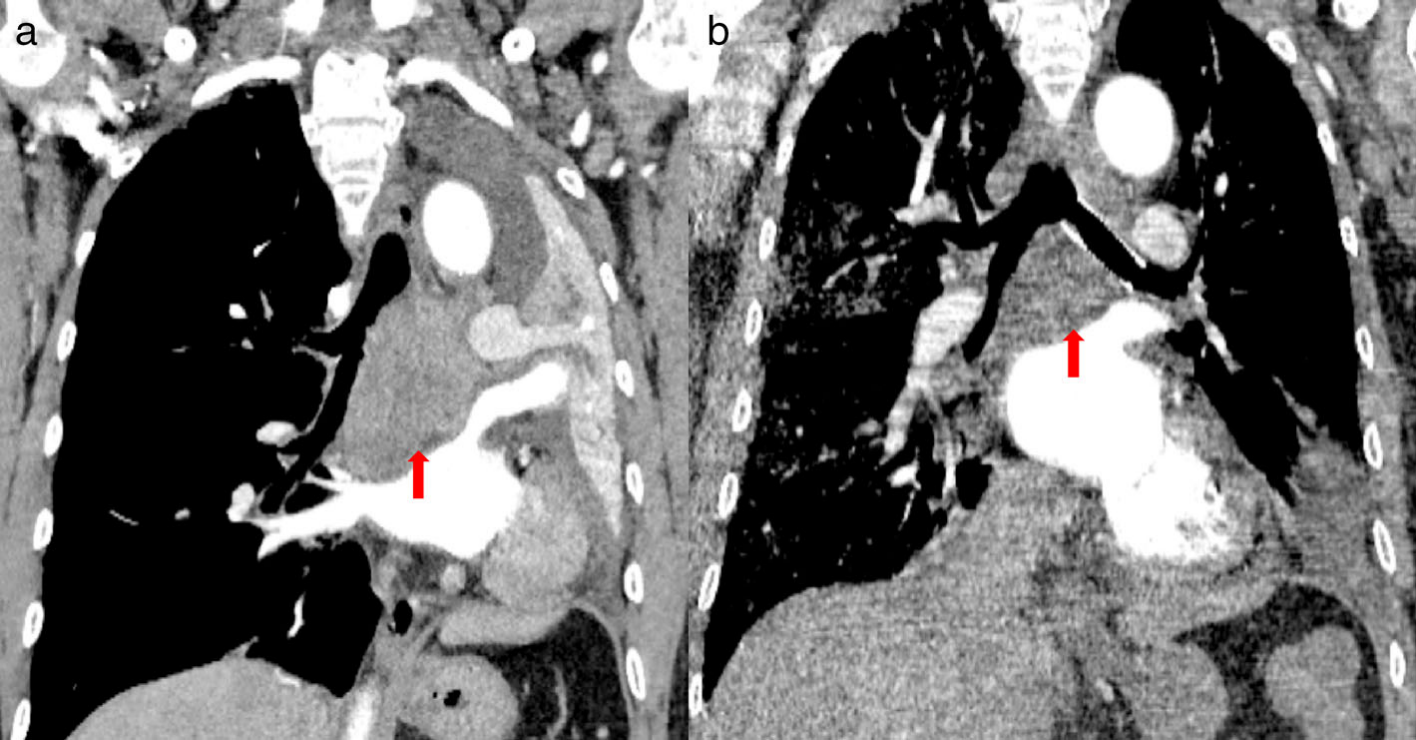
The tumor shrank significantly after seven days combination therapy of crizotinib and osimertinib (red arrows **a**, before; **b**, after).

He subsequently received three cycles of chemotherapy with pemetrexed plus carboplatin, with a partial response. Next‐generation sequencing (NGS) of tumor tissue showed an EGFR 19del mutation (mutant allele frequency [MAF], 47.2%), MET amplification (copy number 2.1), and TP53 (MAF, 36.5%) mutation. Gefitinib (250 mg/day) treatment was initiated in August 2017. Disease progression was documented until March 2019. NGS of plasma ctDNA revealed an *EGFR* T790M mutation, and osimertinib (80 mg/day) was administered. The patient did not receive any radiation therapy to the chest. He subsequently visited our hospital with a complaint of dyspnea in April 2020. Chest CT showed lung cancer which had invaded the carina, causing left lung atelectasis (Fig 1a). Bronchoscopic biopsy revealed adenocarcinoma. NGS of tumor tissue showed EGFR 19del mutation (MAF, 79.3%), met amplification (copy number 6.6), and TP53 mutation (MAF, 53.5%) without *EGFR* T790M mutation. Interventional rigid bronchoscopy was performed to install covered self‐expandable metallic stents and relieve dyspnea in the patient. Postoperative chest radiography showed left lung re‐expansion. Crizotinib (250 mg/twice per day) was initiated. Two days later, he presented with mild dyspnea. Chest CT showed mild bilateral pleural effusion and mild ground‐glass opacities (GGOs) in the right lung (Fig 1b). Seven days later, his dyspnea progressed with respiratory failure. Acute pulmonary embolism was excluded by CT pulmonary angiogram but moderate bilateral pleural effusion, diffuse severe bilateral GGO infiltration and mild consolidation in both lungs were found (Fig 1c). The patient had no peripheral edema, no crackles in the lungs, brain natriuretic peptide was mildly elevated, and echocardiography indicated a normal left ventricular ejection fraction. The bronchoalveolar lavage fluid did not reveal pathogenic microorganisms (bacterial and fungal culture negative, acid‐fast bacilli negative, *pneumocystis jiroveci* negative), and transbronchial biopsy of the right lung showed organizing pneumonia (Fig 2). The tumor shrank significantly after seven days of treatment (Fig 3).

Life‐threatening TKI‐induced interstitial pneumonitis was considered. Crizotinib and osimertinib were stopped immediately. Glucocorticoids were initiated at 80 mg, and the dose was tapered over two months. After the symptoms had been relieved, pemetrexed and carboplatin chemotherapy was administered. Six weeks later, CT scan showed a remarkably absorbed diffuse GGO (Fig 1d). The patient in this study is currently under follow‐up.

## Discussion

Elevated MET copy number amplification in this patient may be a resistance mechanism to EGFR‐TKI. Combination therapy has been reported to have a better overall response rate (ORR), progression‐free survival (PFS) and overall survival (OS) in patients than monotherapy,^1^, ^2^ although there have been some successful reports of crizotinib monotherapy in *EGFR*‐mutant NSCLC that acquired MET amplification.

In this study, we report a case of early‐onset life‐threatening drug related interstitial pneumonitis caused by the addition of crizotinib to osimertinib. Interstitial pneumonitis occurred within two days which was confirmed by CT scan. The incidence of interstitial pneumonia caused by EGFR‐TKI drugs has been previously reported to be 1.1%–1.6%, and the incidence of cases above grade 3 was 0.49%–0.9%.^3^, ^4^ However, this is significantly higher in the Japanese population. The mechanism behind the pulmonary toxicity of these drugs is unclear. EGFR is expressed in type II alveolar epithelial cells and is involved in alveolar wall repair. EGFR‐TKIs may interfere with the alveolar repair mechanism. In previous reports, the onset time of interstitial lung disease (ILD) with erlotinib ranged from 4–47 days, while that of osimertinib ranged from 17–230 days. The median onset of ILD from the initiation of crizotinib therapy was 23 days (range: 3–763 days).^5^ The EGFR and ALK dual inhibitor brigatinib reportedly induced similar early‐onset drug related interstitial pneumonitis.^6^ Supplemental oxygen and systemic glucocorticoids are usually needed. Rechallenge of TKIs after acute lung injury is controversial. Clinicians should therefore be cautious of the potential side effects, particularly early onset interstitial pneumonitis, when using two TKI drugs.

## Disclosure

The authors report no conflicts of interest in this work.
